# Designing Optimized Multi-Species Monitoring Networks to Detect Range Shifts Driven by Climate Change: A Case Study with Bats in the North of Portugal

**DOI:** 10.1371/journal.pone.0087291

**Published:** 2014-01-27

**Authors:** Francisco Amorim, Sílvia B. Carvalho, João Honrado, Hugo Rebelo

**Affiliations:** 1 CIBIO/InBIO, Research Center in Biodiversity and Genetic Resources, University of Porto, Vairão, Portugal; 2 Department of Biology, Faculty of Sciences of the University of Porto, Porto, Portugal; 3 School of Biological Sciences, University of Bristol, Bristol, United Kingdom; University of Western Ontario, Canada

## Abstract

Here we develop a framework to design multi-species monitoring networks using species distribution models and conservation planning tools to optimize the location of monitoring stations to detect potential range shifts driven by climate change. For this study, we focused on seven bat species in Northern Portugal (Western Europe). Maximum entropy modelling was used to predict the likely occurrence of those species under present and future climatic conditions. By comparing present and future predicted distributions, we identified areas where each species is likely to gain, lose or maintain suitable climatic space. We then used a decision support tool (the Marxan software) to design three optimized monitoring networks considering: a) changes in species likely occurrence, b) species conservation status, and c) level of volunteer commitment. For present climatic conditions, species distribution models revealed that areas suitable for most species occur in the north-eastern part of the region. However, areas predicted to become climatically suitable in the future shifted towards west. The three simulated monitoring networks, adaptable for an unpredictable volunteer commitment, included 28, 54 and 110 sampling locations respectively, distributed across the study area and covering the potential full range of conditions where species range shifts may occur. Our results show that our framework outperforms the traditional approach that only considers current species ranges, in allocating monitoring stations distributed across different categories of predicted shifts in species distributions. This study presents a straightforward framework to design monitoring schemes aimed specifically at testing hypotheses about where and when species ranges may shift with climatic changes, while also ensuring surveillance of general population trends.

## Introduction

Ecosystems and global biodiversity are facing a decline as a direct and indirect consequence of human actions [1], [2], and we are yet to experience the full impacts of anthropogenic climate change [3]–[5]. Effective conservation depends on our ability to define, measure, and monitor biodiversity change. Biodiversity monitoring programs usually aim at determining population trends and changes in the structure of biotic communities, often in response to environmental change, anthropogenic disturbance, or targeted management actions [6], [7]. The importance of monitoring biodiversity is becoming increasingly recognized, nevertheless many ongoing monitoring schemes have been criticized for not being underpinned by clear objectives, designed to test specific scientific hypotheses or to evaluate the success of conservation actions (e.g. [7], [8]), and also for not addressing the relation between cost and benefit [9]. One of the problems often identified in monitoring arises from data being collected in an *ad hoc* and fragmented way that lacks statistical and/or methodological consistency and therefore does not allow an effective evaluation of relevant conservation questions [10]. Untargeted monitoring can result in years of wasted effort and money [11] and may even fail in acquiring the critical information to improve management options, one of the major purposes of monitoring [9].

In recent years scientists and practitioners have drawn their attention to the importance of improving methods to design monitoring schemes [7], [8], [12]–[14], and it is widely recognized that monitoring programs need to address well-defined and testable questions, understand how the focal ecosystem might work or how the monitoring targets might function, and be of management relevance (e.g. effects of a pollutant or changes in climate on features of a given ecosystem). However, effective multi-species monitoring is still lacking and, despite its value for describing conditions and detecting undesirable changes, traditionally it is not designed to determine the causes of such changes nor to track specific expected changes [15].

Criticisms on current monitoring programs are particularly evident in the case of monitoring biodiversity responses to climate change. Species may respond to human induced climate change in a variety of ways [16]. Changes in phenology, such as timing of flowering or breeding which may also lead to mismatches between the successive trophic levels [17], have been linked to climate change [18], [19]. An impressive number of studies have also focused on the impact of climate change on species range shifts, from polar latitudes to tropical regions and even marine ecosystems (for an extensive review see [16]).

Species range shifts are a major challenge to conservation planning because the spatial patterns of biodiversity are expected to change, with species of high conservation interest moving from current protected areas (e.g. [20], [21]), while areas that are without legal protection may become more relevant for species’ conservation in the future [22], [23]. Unfortunately, monitoring programs normally disregard where (and when) species ranges are predicted to shift, and thus they are not necessarily designed for detecting shifts due to climate change, although the importance of doing so has been recently acknowledged [24]. Because many species are predicted to be affected by climate change, these programs should also be optimized to monitor the highest number of species for the least cost, something which is rarely considered in the design of the monitoring schemes (but see [10], [15], [25]).

Due to their high mobility, bats are able to respond rapidly to environmental changes [26] rendering them as a good model to detect changes in species distributions due to climate change. In fact changes in the distribution and abundance of bats in response to climate change are already emerging. In Costa Rican cloud forest, bat species have shown an altitudinal shift from lowland areas to higher altitudes in response to climate change [27]. In Europe, *Pipistrellus kuhlii* has expanded its range northwards since the 1990s, in response to the increasing temperatures [28]; and *Pipistrellus nathusii* has also expanded its range towards higher latitudes in the U.K. [29].

Apart from their rapid response to environmental changes, there is high potential for developing bat monitoring programs, as shown by the high number of monitoring programs running worldwide [30], [31]. Currently, the monitoring of cave-dwelling bat species is implemented in several countries (e.g. [32]). However, because surveying tree and crevice-dwelling species can be challenging, time consuming and expensive, monitoring of this group is seldom established. For these species, acoustic sampling is the most widespread method whenever a monitoring program is developed (e.g. [33]–[37]), although some limitations exist e.g. the level of activity is not necessarily proportional to abundance. Moreover factors such as differences in detectability and even temporal variations may lead to differences in species activity [38]. We are fully aware of the inherent limitations of this method, therefore we focused our monitoring networks on species with a higher detection probability.

A good example of acoustic monitoring programs is The Indicator Bats Program (http://www.ibats.org.uk/), with projects running in the UK, Eastern Europe, Ukraine, Russia and Japan. Conversely, other ongoing acoustic monitoring programs have methodological problems, mainly related to sampling design. For example, the Irish Bat Monitoring Program includes a car-based scheme that reveals information on bat populations and distributions [39]. While recognizing the importance of such information, one must be aware that road-based surveys have the potential to provide biased results, since their placement is non-random [15]. Bat monitoring programs have detected population fluctuations (see for example, the 2011 report of the UK National Bat Monitoring Program; available at http://www.bats.org.uk/), but even the best examples are subject to the most frequent problem of monitoring programs (see above): they are not designed to test which environmental changes (e.g. climate change) are leading to such fluctuations.

Predictive models, and particularly species distribution models, allow extrapolating species distribution data in space and time, based on a statistical model [40]. Such extrapolation is possible by combining observations of species occurrences with environmental variables known to influence habitat suitability and therefore species distribution. Combined with stratifications and scenarios for the relevant environmental factors, species distribution models thus have the potential to improve the spatial design and cost-efficiency of ecological monitoring networks (e.g. [41]).

The main goal of this study is to develop and test a framework to design optimized multi-species monitoring networks, able to test hypotheses about how species ranges will shift with climate change. To that end, we developed a case study based on seven bat species in the North of Portugal. We first increased the existing data about bat distribution in the study area with field work targeted at the main gap areas. Next, we used these data to predict likely suitable areas for each species under current and future climatic conditions, and identified, for each species, which areas are more likely to lose, gain or maintain climatic suitability in the future. Then, we used computational tools to optimize the allocation of monitoring stations in space. Finally, we tested whether monitoring networks designed when accounting for predicted species shifts have increased performance relatively to the ones where only the present distributions are accounted.

This study was developed within the scope of SIMBioN – Biodiversity Information and Monitoring System for the North of Portugal, a joint venture between governmental nature conservation agencies and research centers. SIMBioN was designed with the general purpose of regularly providing information on the status of regional biodiversity to support management actions, technical and political decision-making, regarding biodiversity management and conservation. The developed monitoring network should be especially sensitive to biodiversity changes occurring in native woodland areas, for that reason our research focused on the development of a monitoring program for bat species somehow associated with this native habitat type. Because the implementation and running of this network is expected to be fully executed by volunteers, several alternative networks were designed to offer different scenarios considering an unpredictable volunteer commitment. Thus, the main aim of this study is to develop the aforementioned monitoring networks always considering the logistic limitations of volunteer surveyors.

## Materials and Methods

### Ethics Statement

Data on species location used in this research are part of the database from Instituto da Conservação da Natureza e Florestas (ICNF) (the national authority for nature conservation and wildlife protection), and was collected following all legal requirements. Additional data collection within the scope of this research was accomplished using non-invasive methods that do not require legal permits. Sampling in Natural Protected Areas was done with the authorization of ICNF, and samples within private land were performed with the authorization of the land owners.

### Overview of the analytical framework

**Figure 1:**
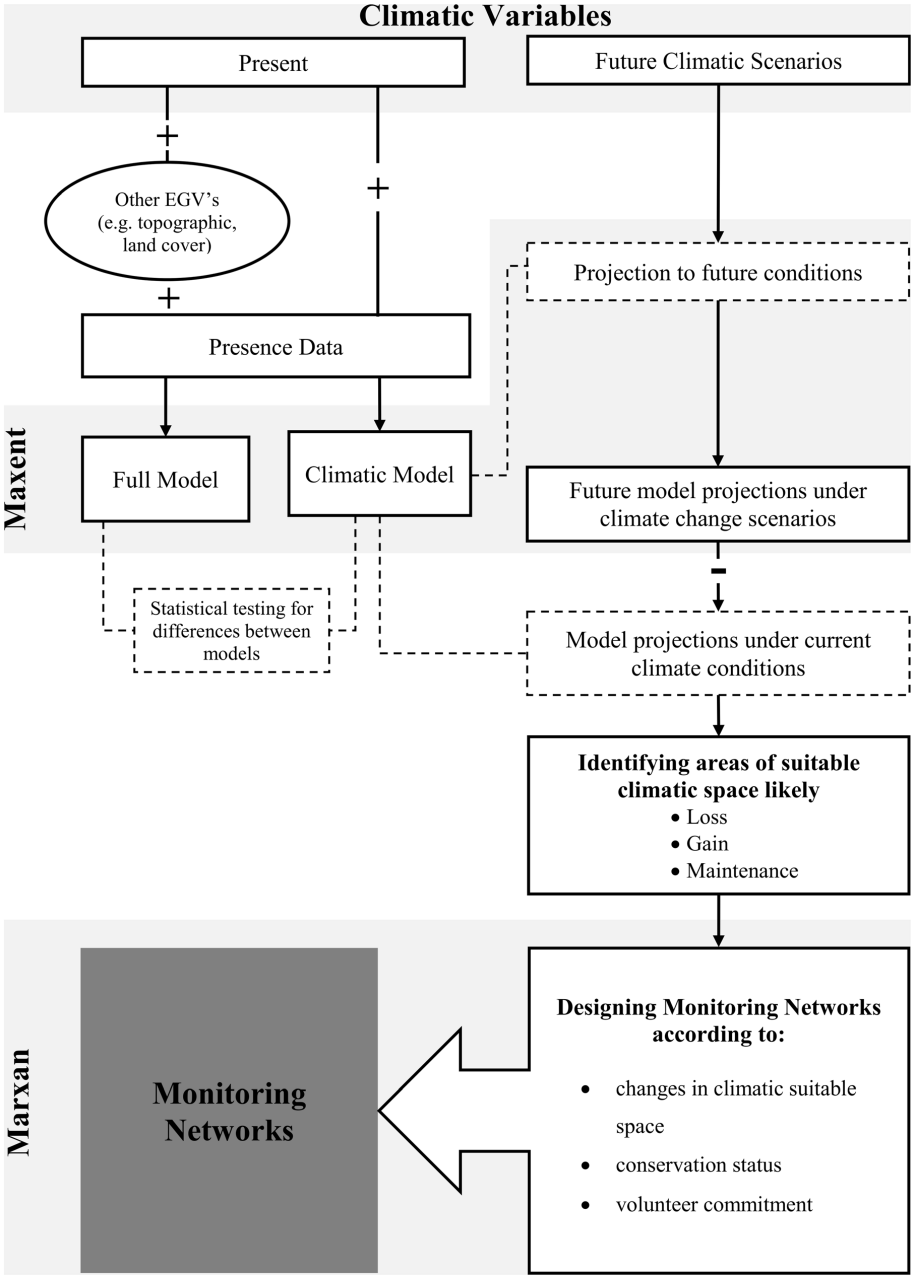
Framework for designing monitoring networks sensitive to climate changes. Proposed framework scheme for designing adaptive monitoring networks sensitive to climate changes. Full lines indicate data and outcomes; dash lines indicate intermediate steps. Data, variables and model addition (+) and subtraction (–) is identified.

Because our framework has many different steps we present here a brief overview of methods, which can also be found in Fig. 1. In a nutshell, using Species Distribution Models we modelled the current distributions of seven bat species and then projected the results to future conditions according to two different climatic scenarios. The difference between current and future predicted distribution allowed us to identify areas where loss, gain or maintenance of suitable climatic space is more likely to occur. These results were then used in combination with conservation planning tools to optimize the location of stations for multi-species monitoring, considering areas where the three different results (loss, gain, maintenance) are more likely to occur.

### Study area

**Figure 2:**
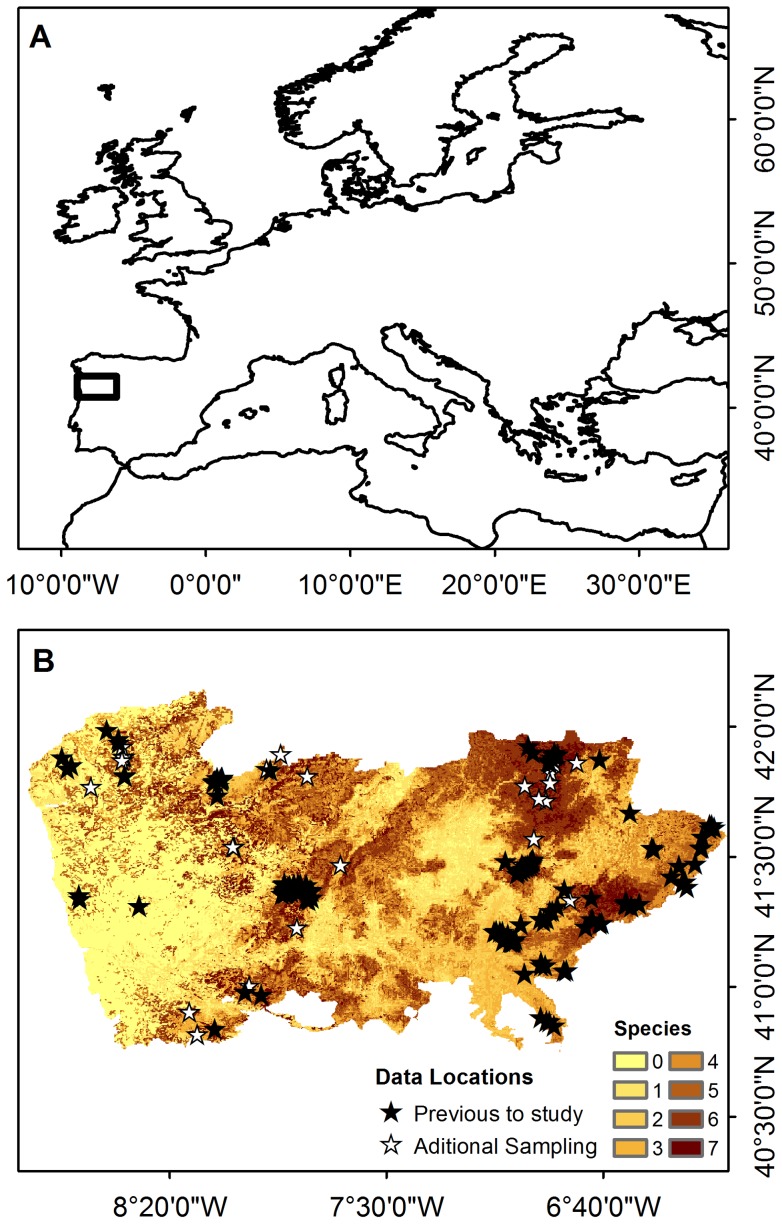
Study area and data locations. Location of the Study area (A) and of the available data before this study (source: Instituto da Conservação da Natureza e Florestas) and additional sampling determined by species richness as predicted by preliminary SMD for the present (B) (see methods).

The study area is located in northern Portugal (Western Europe), approximately between coordinates 40°N–42°N and 6°W–9°W (Fig. 2A). In the northwest and in high elevation areas of the northeast, the Atlantic temperate climate dominates, with mild summers and cold, rainy winters. The landscape is mountainous with native forests mainly composed of deciduous oaks (*Quercus robur*, *Q. pyrenaica*), chestnut (*Castanea sativa*), birch (*Betula celtiberica*), and ash (*Fraxinus angustifolia*). Conversely, in the northeast valleys and lowlands the climate is typically Mediterranean sub-continental, with perennial oaks (*Quercus suber*, *Q. ilex*) dominating the native woodlands.

### Modelling Procedure

For the calculations of species distribution models we chose a presence-only technique, based on the principle of maximum entropy (implemented in Maxent, [42]). The choice of using Maxent over other modelling techniques was based on its very good predictive ability when compared with other methods [43]–[45]. Also, the use of presence-only data is an advantage when reliable absence data are not available or are difficult to assess. Such is the case of bats because of their elusive and nocturnal behaviour [44]. Maxent estimates the range of a species with the constraint that the expected value of each ecogeographical variable (EGV; see below) (or its transform and/or interactions) should match its empirical average (i.e., the average value for a set of sample points taken from the target species distribution [46], [47]). Models were run with 80% of the presence data while the remaining 20% were used for model testing. Because Maxent randomly chooses which presence data to include in the training or test models, we ran 100 model replications and averaged them into a single model. Model calculations were done in the *autofeatures* mode with a maximum of 1000 iterations and the regularization multiplier set to 0.2. To check which variables were the most important to build the model, a Jacknife analysis of the gain was made with the presence data. Jacknife analysis measures how well an EGV distinguishes localities where the species occurs from the total area under study. All calculations were made in Maxent v.3.3.3k.

To forecast the effect of climate change, models were computed with climatic variables only. This may cause overestimations of species occurrence because the distribution of bats may be particularly constrained by land cover. Water habitats such as rivers and ponds can support high levels of bat activity [35], [37] while native woodlands (i.e. oak forests) can support a high bat diversity [36], [37]. To test the effect of this overestimation, we ran two models for the present conditions, one using all the EGVs including land cover, slope and altitude (hereafter “full model”) and one using only the climatic variables (hereafter “climatic model”). To compare these models’ predictions, we used ENMTools v1.3 [48] to measure niche breadth [49] and niche similarity to determine niche overlap [50]. We also checked the percentage of cells in which predictions from both models were in accordance by comparing the cells where presence or absence was predicted. The climatic models calibrated with present climatic variables were projected for future (2080) climatic conditions.

To determine the spatial patterns of current suitable areas for each species, as well as the likely gain and loss of suitable climatic area, all model projections were reclassified into binary presence/absence maps.

### Presence Data and Environmental Variables

The studied bat species were selected according to the availability of occurrence data for the study area and their level of association to native woodland. To achieve reliable models we only considered species with more than 15 records for the study area [51]. Therefore, for model calculations we used as response variables the known locations within the study area for seven bat species: *Myotis daubentonii*, *Pipistrellus kuhlii*, *Hypsugo savii*, *Eptesicus serotinus/isabellinus*, *Nyctalus leisleri*, *Barbastella barbastellus* and *Tadarida teniotis* (source: Instituto da Conservação da Natureza e Florestas; Fig. 2B). Data were collected by several surveyors using different methods (i.e. mist-netting, acoustic sampling and direct observation). Species locations are available in Fig. S1.

In order to increase knowledge about bat distribution in the study area, additional data were collected through acoustic transects. These new surveys were performed in areas of high species richness as predicted by preliminary species distribution models. With this approach some omission errors (false absences) may occur but it allows finding bat species in areas where they were previously unknown while also increasing the geographical coverage of the presence data to be used in subsequent models. Details on the acoustic sampling methods can be found below. A set of independent EGVs, selected as environmental predictors, was considered for model calibration: annual mean temperature (°C), mean diurnal range (°C), mean temperature of warmest quarter (°C), mean temperature of coldest quarter (°C), annual precipitation (mm) (WORLDCLIM; http://www.worldclim.org), altitude, slope (source SRTM; http://www2.jpl.nasa.gov/srtm/), and land cover (Global Land Cover 2005–2006; http://postel.mediasfrance.org/ and Instituto Geográfico Português). Habitat composition and structure is known to influence bat activity, therefore land cover was reclassified into seven ecologically meaningful classes for bats [35]–[37], [52]: urban, agriculture, production forests (mainly pines and eucalypts), scrub and regenerating forest, native woodland, water bodies, and bare ground [44].

Climatic variables (which include all the mentioned EGVs with the exception of altitude, slope and land cover) were chosen according to their reported relevance for bat physiology and survival [53]–[55]. After preliminary tests for the selection of the most informative variables (i.e. the ones achieving higher percentage contribution and gain in model calculations), some of the variables were excluded (max temperature of warmest month, min temperature of coldest month, and precipitation of driest month).

To forecast the effect of climate change on predicted distributions, two contrasting IPCC scenarios (A2a and B2a; http://www.worldclim.org) based on the Global Circulation Model HadCM3 were used. Scenario A2a is driven by economic growth at a regional scale, while B2a considers a regional steady growth and social awareness of environmental sustainability [56]. We used monthly averages of maximum and minimum temperatures and total precipitation, for the period of 2070–2099 (hereafter 2080), and then we calculated the bioclimatic variables according to Hijmans et al. (2005) [57] using the DivaGIS software version 7.5 (www.diva-gis.org).

Altitude, land cover and slope had a spatial resolution of approximately 280x280m. Since climatic variables had a resolution of approximately 1x1km, we downscaled these data to match the cell size of the previous EGVs following the methodological approach of Waltari *et al*. [58]. The study area thus included 494 700 cells, for a total extent of 21 940 km^2^.

### Additional sampling for presence data

In order to increase the quality of data on bat distribution within the study area and add more occurrence data for the target species to our models, additional acoustic transects were carried out between March and August 2010. Transects started one hour after sunset and lasted for three hours [37]. Each transect was walked at low speed (ca. 2 km/h) during 30 minutes using a bat detector (D240X, Pettersson Elektronik AB, Uppsala, Sweden) connected to a digital recorder (Zoom H2, Samson Technologies Inc. USA, New York). Files were saved in WAV format; sampling rate 44.1 kHz and 16 bits/sample. Bat vocalizations were analysed using sound-analysis software (BatSound Pro 3.31, Pettersson Elektronik AB, Uppsala, Sweden) with a 1024 pt FFT and Hamming window for spectrogram analysis [36], [37]. Acoustic identification of bat calls was made through comparison with literature on the theme [59]–[61].

### Monitoring networks

The main goal of our monitoring networks is to support the collection of data on presence or absence of the selected bat species throughout the 21^st^ century. By comparing species distributions from different time intervals (i.e., between current and future sampling) it will be possible to test if observed range shifts occur due to gain, loss or maintenance of climatic suitability. Thus, to be able to test such hypothesis, monitoring stations have to be allocated under a stratified design across these different classes of predicted climatic suitability change.

To calculate the class of suitability change (referred to here as areas where loss, gain or maintenance of species climatic suitability may be observed) in each grid cell, current and future model predictions were reclassified into binary presence/absence maps. For that purpose cells with values above the 10th percentile of training presence were considered suitable for the species [44], [62], [63]. The 10th percentile presence value assumes that 10% of presence data may suffer from errors or lack of spatial resolution [63]. This is especially relevant when dealing with datasets gathered by several researchers (or volunteer surveyors) over large time-spans where reliability and precision has probably varied. Subsequently, each grid cell was classified into one of the three climatic suitability change classes in the following way: cells where climate is suitable in the present (1) but not in the future (0) were classified as “likely lose”; cells where climate is suitable in the present (1) and also in the future (1), were classified as “likely maintain”; cells where climate is currently unsuitable (0) but predicted to become suitable in the future (1) were classified as “likely gain”. This classification was done separately for the two different climatic scenarios (A2a and B2a). Because we did not include EGVs such as land cover, slope and altitude to predict future suitability, and acknowledging their major importance for bats (particularly land cover [36], [37], [52]), we ensured that at least one quarter of the monitoring stations fell within areas predicted as currently suitable in the full model. By doing so we enforced that some sampling locations of the Monitoring Networks were set for areas where the values of those EGVs are currently suitable for species occurrence.

The next step consisted of determining the number of monitoring stations to be included at each suitability class for each species. Because the viability of the monitoring program is dependent on an unpredictable volunteer commitment, multiple designs were developed covering different citizen engagement scenarios, with increased number of monitoring stations for each species (MN1, MN2 and MN3). To make it possible to gradually expand the monitoring network without losing any information from previous campaigns, stations from MN1 were included in MN2 and stations from the latter were included in MN3. The number of sampling stations was set based on the level of expected commitment depicted from the levels of participation in bat detector workshops and environmental actions in the study area. Additionally, because different species have different conservation concerns, the monitoring effort allocated to each species was also set as a function of the species conservation status at the National level (for species with higher conservation status, a higher number of monitoring stations was set) [64]. Table 1 shows the minimum number of monitoring stations targeted in each network (MN1, MN2 and MN3) for each species, likely occurrence class, and full model.

**Table 1:** Minimum number of locations targeted in each monitoring network (MN1, MN2 and MN3) for each species suitability class (G – Likely Gain; M – Likely Maintain; L – Likely Loss) and for areas predicted as currently suitable in the full model (Full).

	Conservation	MN1				MN2				MN3			
	Statuts	G	M	L	Full	G	M	L	Full	G	M	L	Full
Mdau	LC	3	3	3	3	6	6	6	6	12	12	12	12
Pkuh	LC	3	3	3	3	6	6	6	6	12	12	12	12
Hsav	DD	5	5	5	5	10	10	10	10	20	20	20	20
Nlei	DD	5	5	5	5	10	10	10	10	20	20	20	20
Eser/isa	LC	3	3	3	3	6	6	6	6	12	12	12	12
Bbar	DD	5	5	5	5	10	10	10	10	20	20	20	20
Tten	DD	5	5	5	5	10	10	10	10	20	20	20	20

Species code as follow: Myotis daubentonii (Mdau); Pipistrellus kuhlii (Pkuh); Hypsugo savii (Hsav); Nyctalus leisleri (Nlei); Eptesicus serotinus/isabellinus (Eser/isa); Barbastella barbastellus (Bbar) and Tadarida teniotis (Tten).

We used the software Marxan [65] to identify optimized sets of monitoring stations to track range shifts in multiple bat species. Marxan is a decision-support tool which uses a simulating annealing algorithm to minimize the amount of selected sampling units whilst ensuring the representation of a set of features (species, habitats, or other features) with a given minimum number of occurrences (occurrence target) [66]. Marxan was conceived to assist decisions about the location and design of protected area systems, but the mathematical problem underlying the optimization of monitoring network is very similar. In our case, the conservation features that we want to represent are the three classes of suitability shift for each species plus the suitable areas predicted under the full model. Reserve selection problems can also incorporate aggregation and connectivity rules which are not desirable when designing monitoring networks, because the further apart monitoring stations are, the more independent the monitoring data will be. Marxan was configured with the following parameters: algorithm – simulated annealing; number of runs – 100; penalty cost for not achieving the occurrence target – 100; iterations per simulation – 1,000,000; temperature decreases per simulation – 10,000; initial temperature and cooling factor – adaptive. For MN2, the status of the grid cells selected in the best solution of MN1 was set to 2 in order to force MN2 solution to include monitoring stations selected in MN1. This would simulate an expansion of the monitoring network as citizen engagement increases. We followed the same procedure for MN3 with the grid cells selected in the best solution of MN2. No boundary length modifier was used. Targets were set as defined in Table 1.

### Performance of optimized vs. non-optimized networks

Three additional networks were designed to test whether our proposed framework has potentially increased performance in detecting species range shifts derived by climate change than the commonly used approach which only considers the current distribution of the species. For this purpose, we have rerun Marxan setting targets only for current predicted distribution of each of the seven bat species for climatic and full model. Target values were set according to the number of sample stations in each of our three monitoring networks that fall within current predicted suitability considering climatic and full model.

We used Cost Threshold function in Marxan to limit the overall number of sampling stations so that it would be equal to the number of stations selected in each of our three monitoring networks, respectively 28, 54 and 110. Differences between the performance of our framework and the testing networks were accessed by checking the proportion of the targets set in Table 1 that were not met in each of the 100 runs of the testing networks.

## Results

### Additional sampling for presence data

A preliminary set of species distribution models calibrated using the presence data for the targeted species available prior to this study allowed us to set 35 additional sampling sites in areas predicted to have high species richness (Fig. 2B). As a result of in-field campaigns, 418 new bat passes were recorded, adding 21 new locations for the targeted species. Species locations and presence/absence maps predicted by preliminary models are available in Fig. S1.

### Current bat diversity patterns and ecological predictors

Regarding the predictive ability of the full model, test data showed only slightly lower values for AUC (area under the receiver operating characteristic curve, which ranks all locations according to their suitability [42], [67]) than training data (AUC for test data ranged 0.78–0.86, AUC for training data ranged 0.86–0.93), which is an indication that no over-fitting occurred in the models. The high values of test AUC also indicate a good transferability power of the model. Likewise, climatic models showed good predictive power, with AUC ranging from 0.82–0.91 in the training data and 0.76–0.83 in the test data. For more details on the AUC values for each species see Table S1.

In the full model, land cover, altitude, annual mean temperature and temperature of coldest quarter were the most relevant EGVs for the majority of species (Fig. S2). When model fitting was based on climatic variables only, temperature of coldest quarter, temperature range, annual precipitation and annual mean temperature were the most relevant for most species (Fig. S3). A more comprehensive list of relevant predictor variables for each model and species (Table S2), as well as the corresponding response curves (Fig. S4-Fig. S10), can be found in Supporting Information.

**Figure 3:**
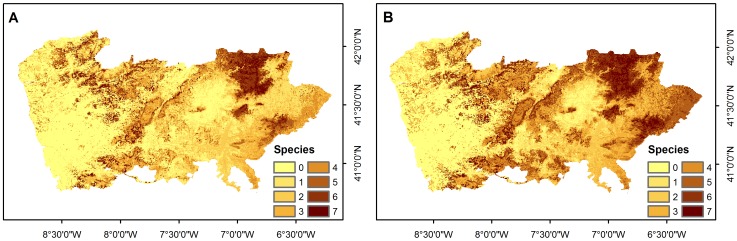
Current predicted species richness for full model (A) and climatic model (B). Current predicted species richness for full model (A) and climatic model (B).

Predicted species richness showed similar spatial patterns in the full models and in the climatic models (Fig. 3). In fact, binary predictions from both models spatially overlapped in more than 70% of the total cells for all species. Climatic models alone predicted suitability in at least 12% more cells, whereas the full model predicted unique suitable sites in less than 8% of the area (Table S3). Results from the niche overlap and *D* statistics ranged 0.95–0.99 and 0.78–0.88 respectively, thus confirming similar predictions between full and climatic models (Table S3). Regarding niche breadth metrics, results showed that the full model yielded a narrower niche (Table S3).

Predicted species richness showed some level of spatial structure (Fig. 3), with the majority of the highest values of species richness located in the northeast of the study area where high species richness has a more continuous distribution.

### Future projections

**Figure 4:**
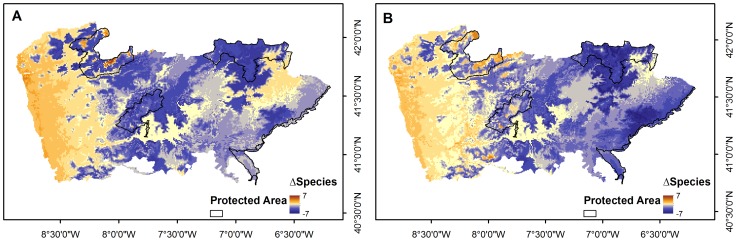
Predicted variation in climatic space between the present and the future. Predicted variation in climatic space for target species between the present and the future under climatic scenarios A2a (A) and B2a (B) for the year 2080. Negative ▵Species values indicate loss of climatic suitable space while positive indicate species gain of climatic suitable space. Most important protected areas are also represented.

Overall, bats in the study area have a high sensitivity to climatic changes, resulting in large extents of potential loss of climatic suitability (Fig. 4). When comparing current predicted distribution of species for both climatic scenarios, the area which could lose climatic suitability in the future for at least one species represents more than 60% of the study area (A2a: 62.5%; B2a: 64.2%) and in more than 30% likely loss was predicted for three or more species (A2a: 34.6%; B2a: 37.7%). In contrast, climatic suitability gain for at least one species is only predicted in 10.8% of the area in scenario A2a and 15.4% in scenario B2, while for scenarios A2a and B2a respectively 26.8% and 20.4% may maintain climatic suitability for the target species.

Areas where gain may occur were limited to a narrow fringe along the coastline in the western part of the study area and small isolated strongholds most of which located in the northwest of the study area (Fig. 4). Individual species maps may be found in Fig. S11 and Fig S12.

### Monitoring networks

**Figure 5:**
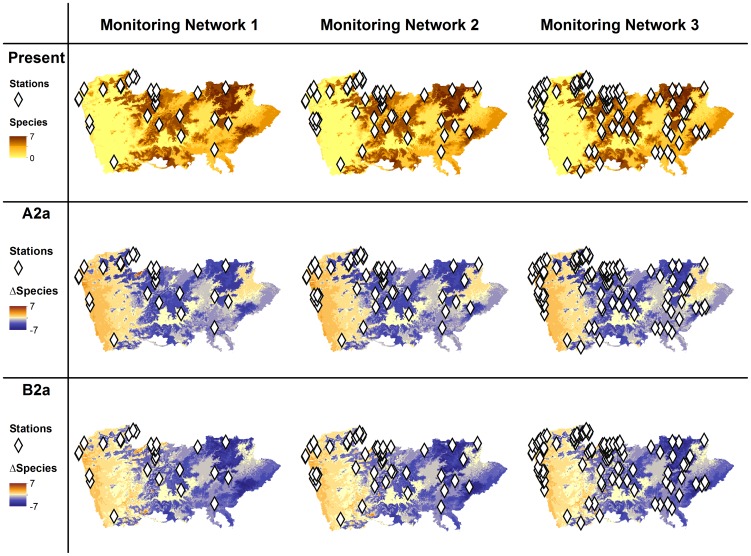
Sampling stations for the proposed monitoring networks (MN1, MN2 and MN3). Sampling stations for the proposed monitoring networks (MN1, MN2 and MN3) showing the present predicted species richness according to climatic model and predicted variation in climatic space between the present and the future under two climatic scenarios (A2a and B2a) for the year 2080. Negative ▵Species values indicate loss of climatic suitable space while positive indicate species gain of climatic suitable space.

The targets set for MN1 were achieved with a total of 28 sites, while for MN2 and MN3 targets were accomplished with 54 and 110 sites, respectively (Fig. 5). In MN2 and MN3 it is possible to observe a concentration of sites in some areas. This happens mainly because there are very few areas where likely suitability gain was predicted in the future scenarios. The general distribution pattern of sites is similar for the three Monitoring Networks, nevertheless, as expected, the higher number of locations in MN2 and MN3 results in an increase of the spatial coverage of the resulting monitoring networks.

### Performance of optimized vs. non-optimized networks

**Figure 6:**
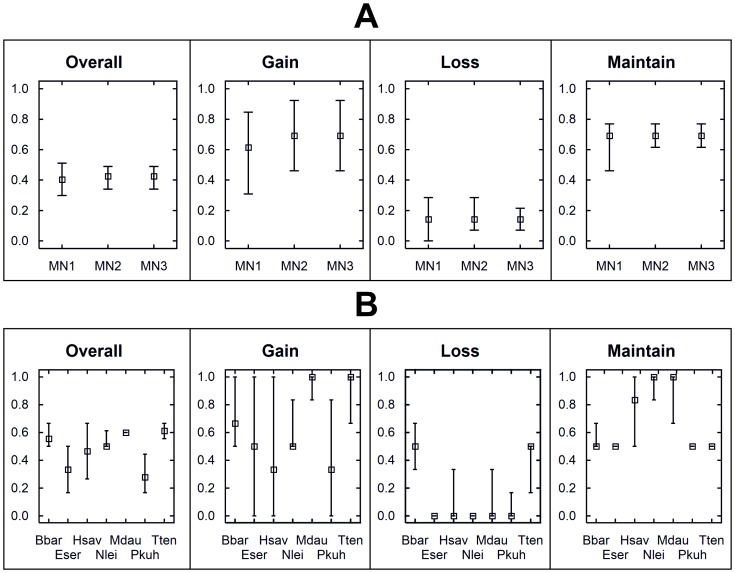
Proportion of targets that were not met in each of the testing networks (A) and by species (B). Proportion of targets that were not met by the 100 runs in each of the testing networks (A) and by species (B) considering overall targets and targets set for each suitability class. Median, maximum and minimum values are presented. Species code as follow: *Barbastella barbastellus* (Bbar); *Eptesicus serotinus/isabellinus* (Eser); *Hypsugo savii* (Hsav); *Nyctalus leisleri* (Nlei); *Myotis daubentonii* (Mdau); *Pipistrellus kuhlii* (Pkuh); and *Tadarida teniotis* (Tten).

None of the 100 Marxan runs for each of the non-optimized networks was able to meet all the targets set in Table 1. Between 30% and 50% of the total targets were not met in either of the networks (Fig. 6A). Failure in achieving the targets was more pronounced in the climatic suitability classes of “likely gain” and “likely maintain” (Fig. 6A). Considering the achievement of targets set for individual species, we observed a large range of results, nonetheless “likely gain” and “likely maintain” were also the most problematic classes, while targets set for “likely loss” of climatic suitability were met by five out of the seven species (Fig. 6B).

## Discussion

### Designing and implementing optimal monitoring schemes under climate change

We presented an innovative approach representing an improvement in the design of monitoring networks that goes beyond the conventional surveillance schemes in the sense that it allows testing hypotheses about how environmental change (in our case, climate change) will drive species distributions. At the same time it produces fundamental data for the surveillance of species and communities at regional scales, which is one of the fundamental goals of biodiversity monitoring [68]. We have shown that, when compared with conventional network designs, the proposed framework has increased performance in allocating monitoring stations distributed across different categories of predicted shifts in species distributions, which is crucial to test hypotheses about the effects of climate change on species ranges. In conventional, non-optimized networks, because only the current distribution of the species was considered, we expected that selected monitoring stations were allocated more or less randomly across the species ranges, only depending on the level of species co-occurrence. Thus, we expected that for species likely to lose a great proportion of their current distribution due to climate change, it would be more difficult to allocate monitoring stations to areas that fall under the “likely maintain” class and vice versa. Our results confirmed this expectation, as most species likely to lose a great proportion of their climatic suitability (Fig. S13) were the ones with lower target achievement in the “likely maintain” class (e.g. Hsav, Nlei and Mdau in Figure 6B). We also expected that the “likely gain” class was the one less represented in the conventional networks, because these areas fall outside of the current distribution of the species. Our results also confirmed this expectation (Fig. 6), although some stations were indeed selected in areas of likely gain of climatic suitability for some species. This fact can be explained by the co-occurrence of two or more species. For instance, a monitoring station may be allocated to represent the current distribution of species A and B, and this location may be, by chance, within the area of likely gain of climatic suitability of species C. Co-occurrence may also explain why targets for the “likely lose” class were achieved for species such as *P. kuhlii* and *E. serotinus*, which have a low proportion of likely loss of climatic suitability.

Our framework also allows adjusting the sampling effort according to a frequently unpredictable volunteer commitment and to prioritize monitoring effort according to each species conservation status. Though we choose not to include the costs of implementing the sampling stations in the suggested locations, we point out that such cost can be considered when using Marxan [69]. The measures of cost can be based on any relative social, economic or ecological cost, or combinations thereof [69]. A critical example when designing monitoring networks based on volunteer effort is the costs of accessibility of different areas, which could be easily incorporated in Marxan by applying a planning unit cost factor.

Although it is highly likely that our simplest monitoring network (MN1) will not allow gathering enough information for a robust analysis, it will accomplish the fundamental goal of having sampling stations in areas where species and community structure are predicted to be sensitive to future climate changes. Moreover, it will work as a pilot survey to evaluate the statistical power of the monitoring network to detect population changes, as it is often suggested in adaptive monitoring [7], [12]. To maximize accuracy and minimize the possibility of biased conclusions being drawn about trends, a power analysis should subsequently be performed, e.g. following Walsh et al. [70]. The evaluation of volunteer efforts and the improvement of the network effectiveness could be fine-tuned following Tulloch *et al*. [71].

Operationally, the implementation of MN1 could allow attracting progressively more volunteers for the program – in fact it should be considered as the first step in the establishment of a more robust monitoring network. By working on fund-raising along with state agencies and NGO’s, and depending on volunteer commitment, the underlying expectation is that the sampling effort will increase in the near future. The experience gathered with the ongoing Portuguese Bat Atlas (http://anodomorcego.wix.com/icnb), and also the example of the Portuguese Breeding Bird Atlas [72], allow some confidence on the growing commitment of citizens. Moreover, the increasing number of advanced courses and free workshops on ultra-sound recording techniques and species identification in Portugal has shown that non-specialists have strongly embraced this type of citizen science activities and transference of skills [73]. Overall these experiences strongly suggest that it is possible to successfully implement a long-term bat monitoring network in Portugal.

### Model predictions and model-based simulations under climate change

Our results show that bats in the study area are highly sensitive to climate change, and though our main goal was not to determine the spatial patterns of future species richness, the findings presented here are in line with studies on the subject. A study of 28 European bat species hypothesized that a major range shift towards northern latitudes (U.K. and Fenno-Scandinavia) will occur until the end of the century, showing a significant loss of species richness in the Iberian Peninsula [74] and particularly in our study area. Other studies including amphibians, reptiles or trees [75], [76] also predict a major loss of species ranges in the Atlantic climatic regions, mostly located along the north and northwest of the Iberian Peninsula. Also, the Scenarios, Impacts and Adaptation Measures (SIAM) report [77] identified the north-eastern and eastern parts of our study area as highly sensitive to climate changes, which is consistent with our results, while Thuiller *et al*. [78] included the northwest Iberian Peninsula among those areas in Europe where climate change would cause highest levels of plant species loss and turnover.

Nonetheless, we should be aware that future species richness is most likely underestimated since our models only used partial information about the environmental niche for the target species. This can be critical when suitable climatic space is projected for future climate scenarios because the truncated niche can be responsible for an over-prediction of local extinctions at southern distribution edges in the northern hemisphere [79], [80]. It is highly probable that southern Iberian populations of the target species could colonize the study area when ecological conditions become unsuitable at their southernmost ranges, compensating for otherwise forecasted local extinctions. Although the approach used here may reduce the models’ applicability for extrapolation purposes, e.g. for predicting species–habitat interactions for other areas, times or climates [81], we stress that our main goal was to determine areas in the region that are sensitive to climate change regarding bat species, and not necessarily to predict the future distributions of the species in the region. In other words, we identified and will monitor areas where changes in species occurrence driven by future climatic shifts are more likely to occur.

In the present study, the simulations did not account for land cover changes in future projections although habitat variables are recognized to be relevant in predicting bat species distribution [36], [37], which is also supported by the results obtained with our full model. Long-term changes in land cover are difficult to predict, especially in highly humanized areas because of the dependence upon economic interests and policy guidelines (among other drivers; e.g. [82]), and the inclusion of such variables would bring a higher degree of uncertainty to future projections. Although we recognize the importance of land cover, we should emphasize that the high agreement in niche overlap statistics show that full models and climatic models have a high similarity in their projections under current conditions. This result, together with the broader niche breadth obtained for the climatic model, means that the latter captures almost all niche conditions of the full model. For that reason, the use of the climatic model for future projections did not compromise our results. Nonetheless, to overcome this potential source of uncertainty, we opted to include a quarter of the sampling stations of the monitoring networks in areas where species occurrence was predicted when modelling with land cover. This approach may allow understanding whether future changes might be due to climate changes and/or to changes in land cover.

By combining different model techniques and circulation models we can achieve more robust projections, significantly reducing prediction uncertainties [83]. Although future predictions of species distributions were not the main scope of this study, these outputs can then be easily incorporated in the proposed framework. This innovative methodology can be used on any taxon or spatial scale, allowing a statistical optimization of the allocation of sampling effort in areas with high biodiversity that are also predicted to be prone to environmental changes.

### Implications for conservation

To our best knowledge, the vast majority of current monitoring networks do not take into account in their design the potential changes in species distributions that may result from future climate change. By producing reliable data to detect population trends and range shifts due to climate change, the monitoring networks proposed here will provide stakeholders with important outcomes for conservation. MN1 will accomplish the immediate goal of attracting volunteers and to set a pilot survey [12]. If needed, future monitoring networks will then be fine-tuned in response to new information or new questions [7]. Power analysis will be performed during the lifetime of the project, a fundamental step because the consequences of inadequate design may not be obvious until the end of the programme, when it may be too late for amendments.

By monitoring climate change sensitive areas it will be possible to identify the most resilient populations which will be paramount for the future conservation of biodiversity. These populations will harbour unique gene pools while being source populations to colonise the new suitable areas. Moreover, it may be possible to understand species movements and consequently design corridors that promote their dispersion (e.g. [84]), focusing conservation and management efforts where they can produce the best results under environmental change scenarios. The implementation of this framework could provide an example for the development of climate change sensitive monitoring networks for other taxa and geographic contexts.

## Supporting Information

Figure S1 **Presence data for each target species and binarized maps of predicted occurrence according to preliminary Species Distribution Model.**
(TIF)

Figure S2 **Full model Percentage contribution of predictor variables and regularized gain With Only and Without predictor variables.**
(TIF)

Figure S3 **Climatic model Percentage contribution of predictor variables and regularized gain With Only and Without predictor variables.**
(TIF)

Figure S4 **Response curves for the EGVs most related to the predicted distribution of** ***Myotis daubentonii*** **.**
(TIF)

Figure S5 **Response curves for the EGVs most related to the predicted distribution of** ***Pipistrellus kuhlii*** **.**
(TIF)

Figure S6 **Response curves for the EGVs most related to the predicted distribution of** ***Hypsugo savii*** **.**
(TIF)

Figure S7 **Response curves for the EGVs most related to the predicted distribution of** ***Eptesicus serotinus/isabellinus*** **.**
(TIF)

Figure S8 **Response curves for the EGVs most related to the predicted distribution of** ***Nyctalus leisleri*** **.**
(TIF)

Figure S9 **Response curves for the EGVs most related to the predicted distribution of** ***Barbastella barbastellus*** **.**
(TIF)

Figure S10 **Response curves for the EGVs most related to the predicted distribution** ***Tadarida teniotis*** **.**
(TIF)

Figure S11 **Areas where each target species is likely to gain, lose or maintain suitable climatic space under scenario A2a.**
(TIF)

Figure S12 **Areas where each target species is likely to gain, lose or maintain suitable climatic space under scenario B2a.**
(TIF)

Figure S13 **Proportion of area occupied by suitability class for each species.**
(TIF)

Table S1 **AUC values for Training and Test data for both Full and Climatic models.**
(DOCX)

Table S2 **Predictor variables by order of relevance for each species for both Full and Climatic models.**
(DOCX)

Table S3 **Percentage of the total number of cells that overlap is observed in both Full and Climatic and ENMtools results.**
(DOCX)
