# Supplementary figures and images for: Designing Optimized Multi-Species Monitoring Networks to Detect Range Shifts Driven by Climate Change: A Case Study with Bats in the North of Portugal
Source: PLoS One. 2014 Jan 27;9(1):e87291. doi: 10.1371/journal.pone.0087291 (PMC3903647; doi:10.1371/journal.pone.0087291)

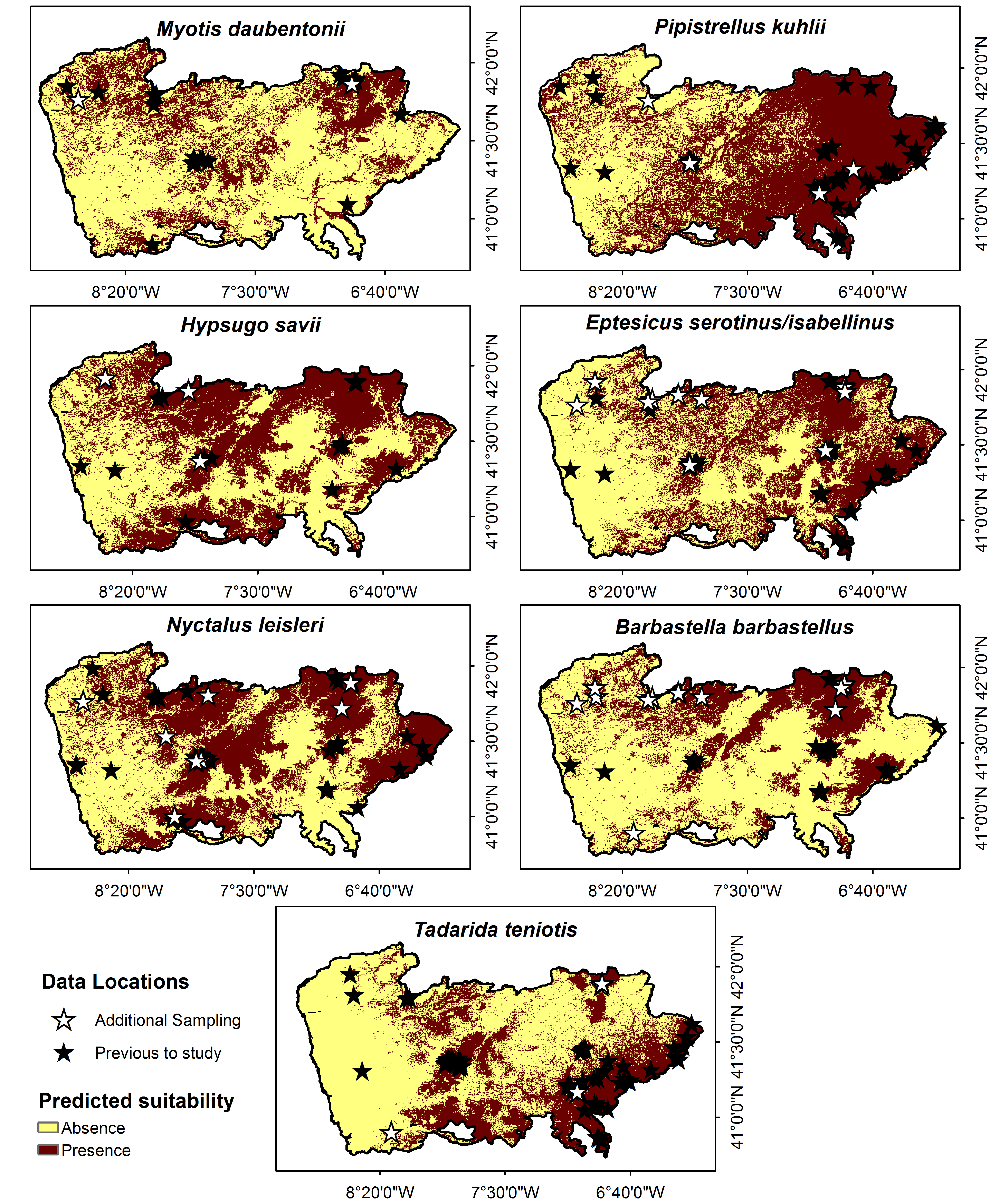

Supplement: Figure S1 — Presence data for each target species and binarized maps of predicted occurrence according to preliminary Species Distribution Model. (TIF) [file pone.0087291.s001.tif]

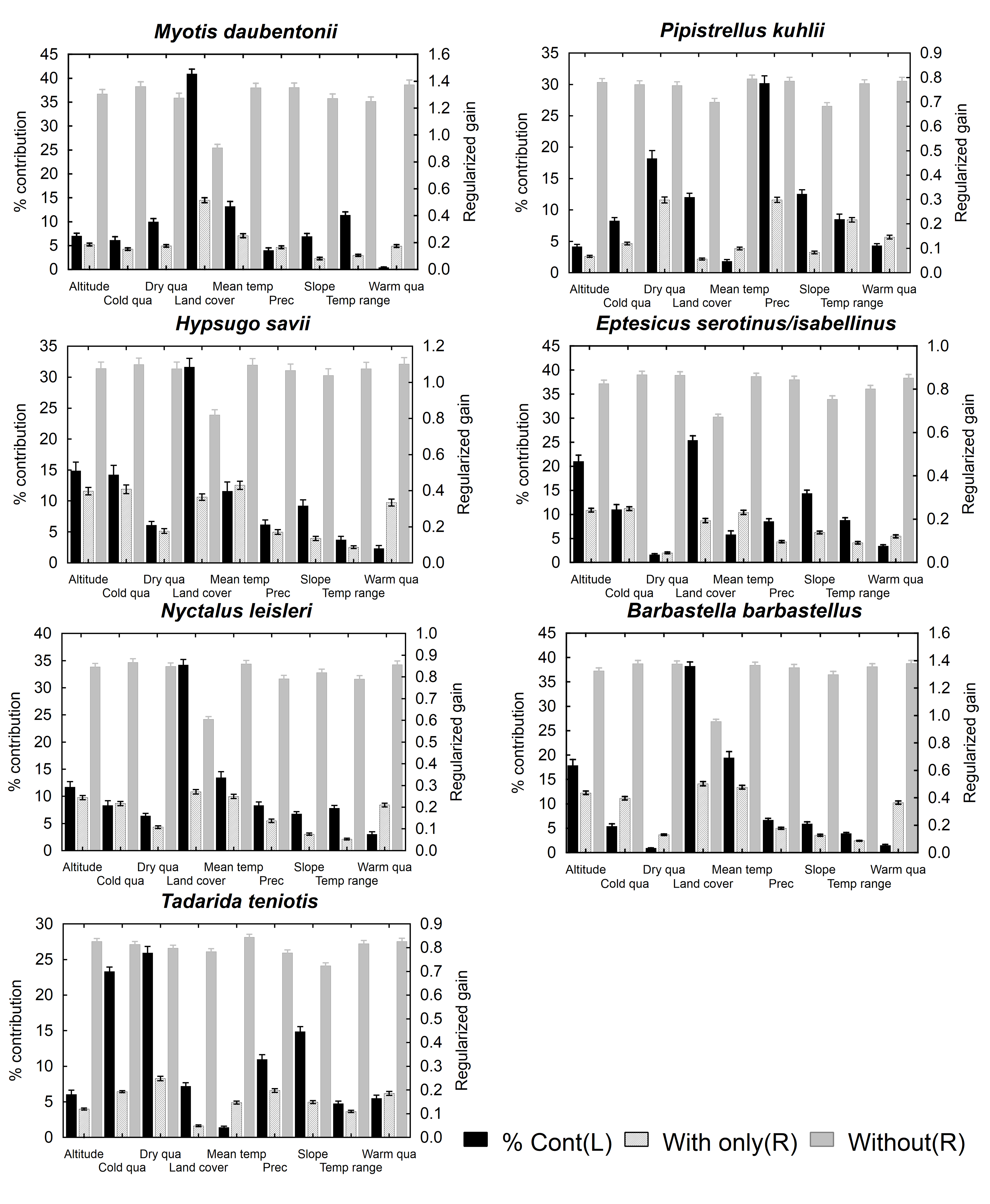

Supplement: Figure S2 — Full model Percentage contribution of predictor variables and regularized gain With Only and Without predictor variables. (TIF) [file pone.0087291.s002.tif]

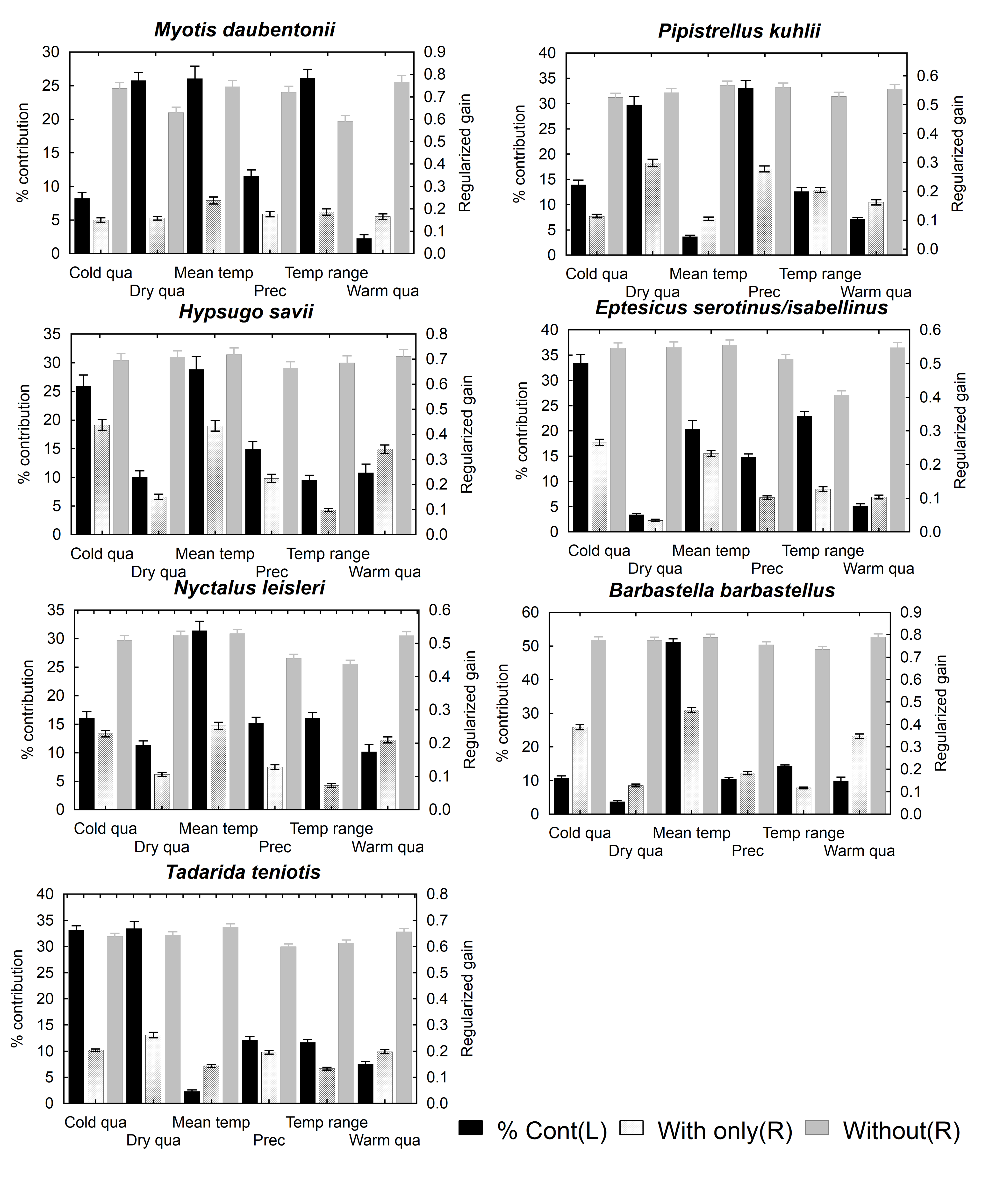

Supplement: Figure S3 — Climatic model Percentage contribution of predictor variables and regularized gain With Only and Without predictor variables. (TIF) [file pone.0087291.s003.tif]

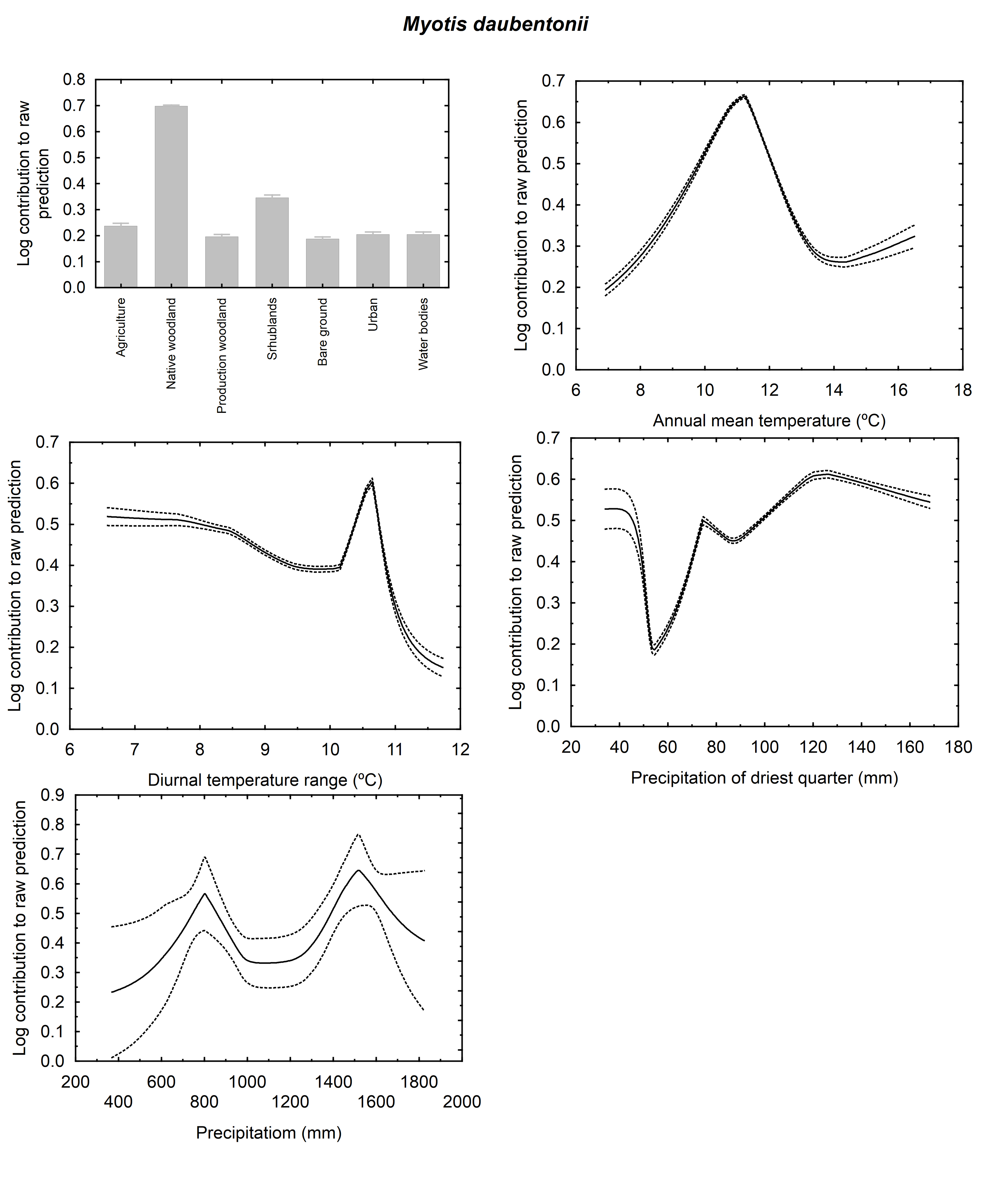

Supplement: Figure S4 — Response curves for the EGVs most related to the predicted distribution of Myotis daubentonii . (TIF) [file pone.0087291.s004.tif]

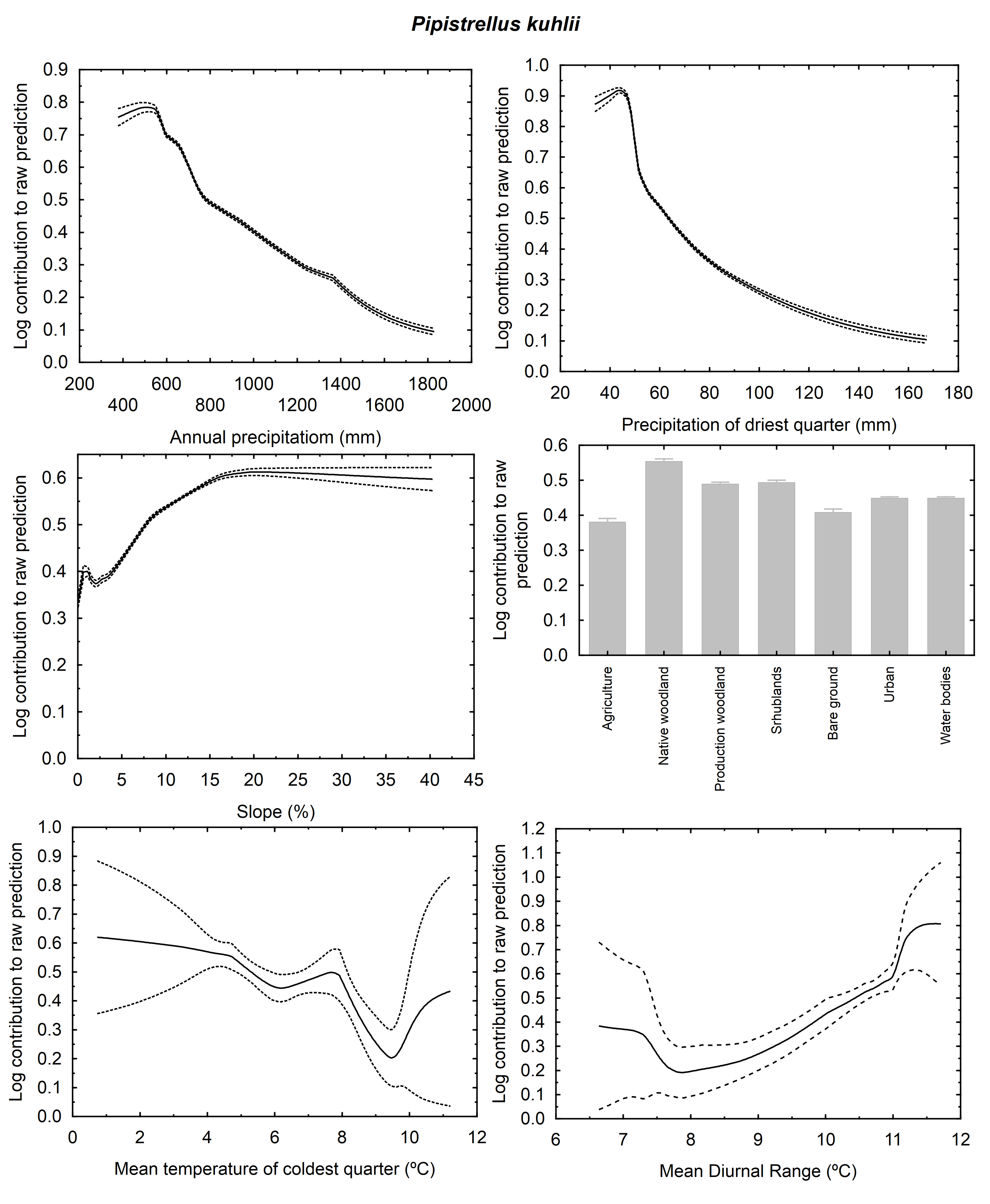

Supplement: Figure S5 — Response curves for the EGVs most related to the predicted distribution of Pipistrellus kuhlii . (TIF) [file pone.0087291.s005.tif]

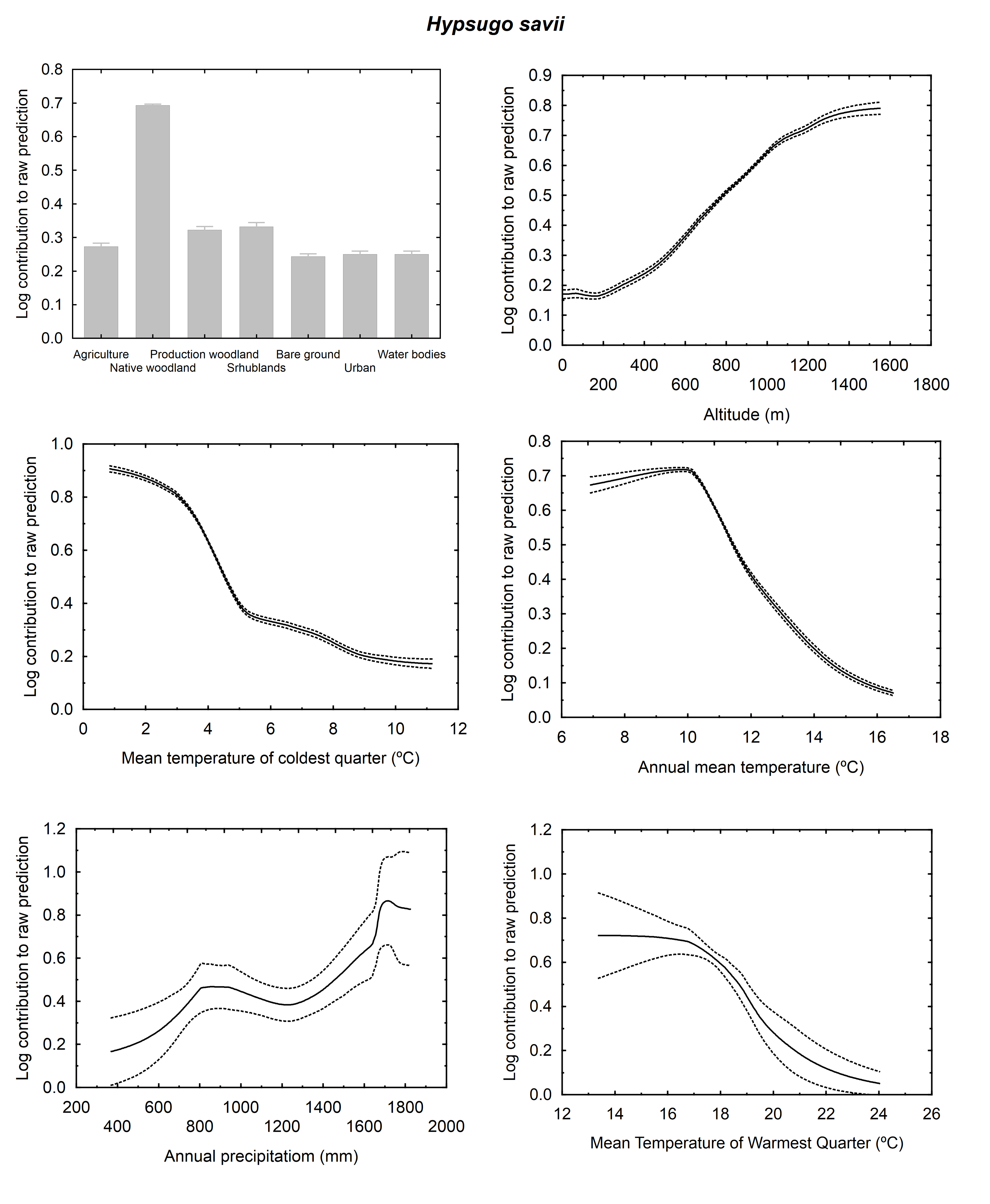

Supplement: Figure S6 — Response curves for the EGVs most related to the predicted distribution of Hypsugo savii . (TIF) [file pone.0087291.s006.tif]

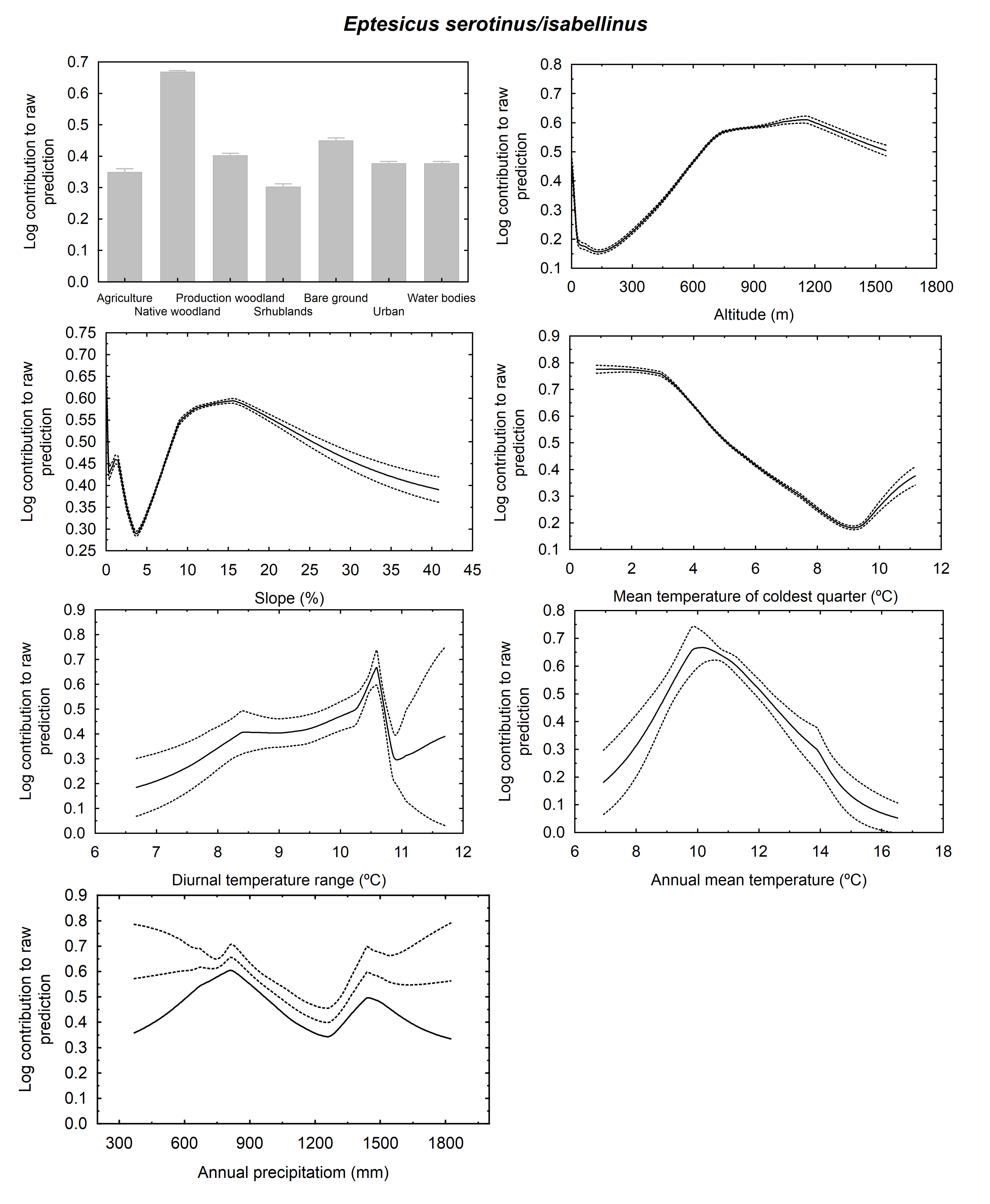

Supplement: Figure S7 — Response curves for the EGVs most related to the predicted distribution of Eptesicus serotinus/isabellinus . (TIF) [file pone.0087291.s007.tif]

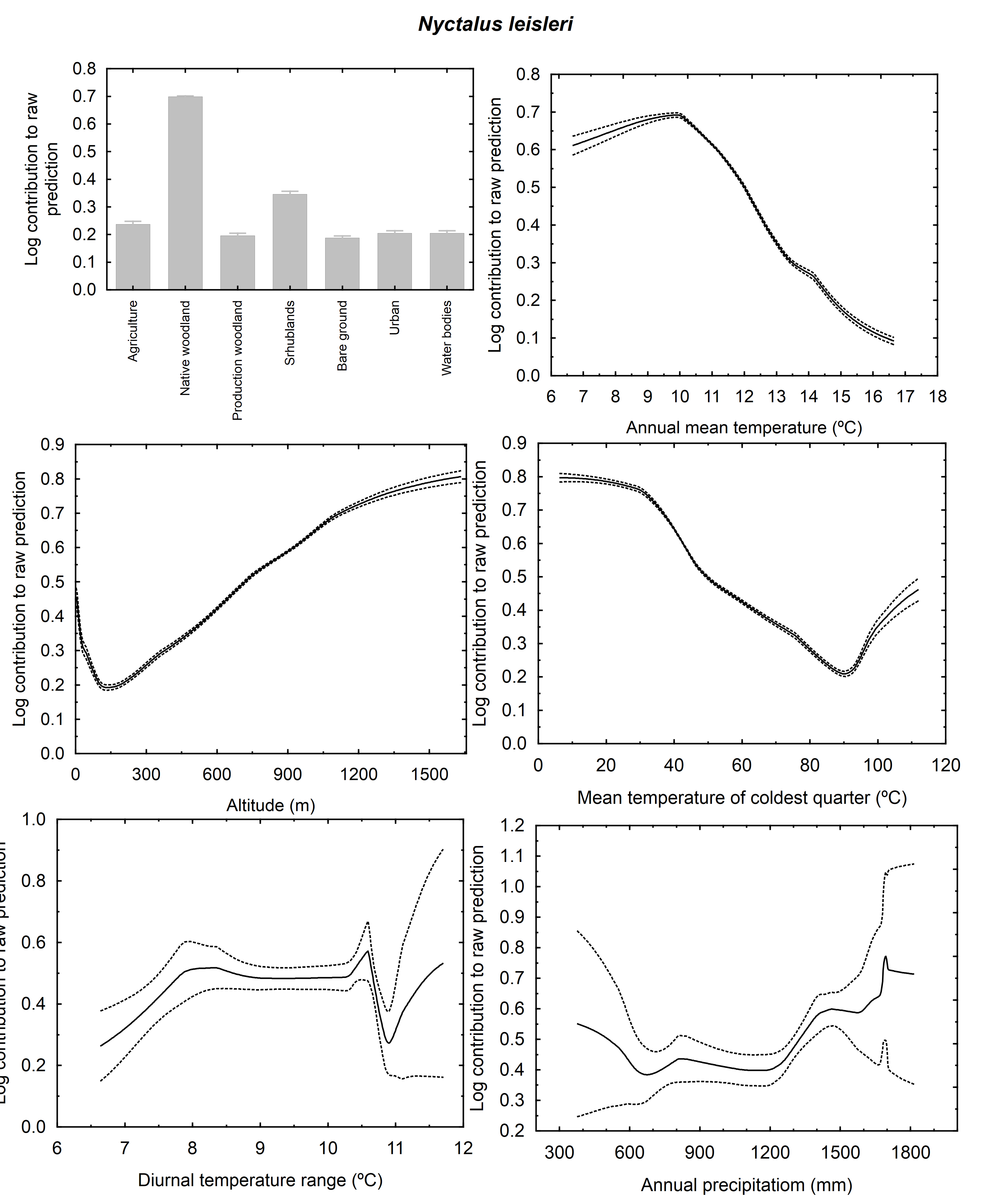

Supplement: Figure S8 — Response curves for the EGVs most related to the predicted distribution of Nyctalus leisleri . (TIF) [file pone.0087291.s008.tif]

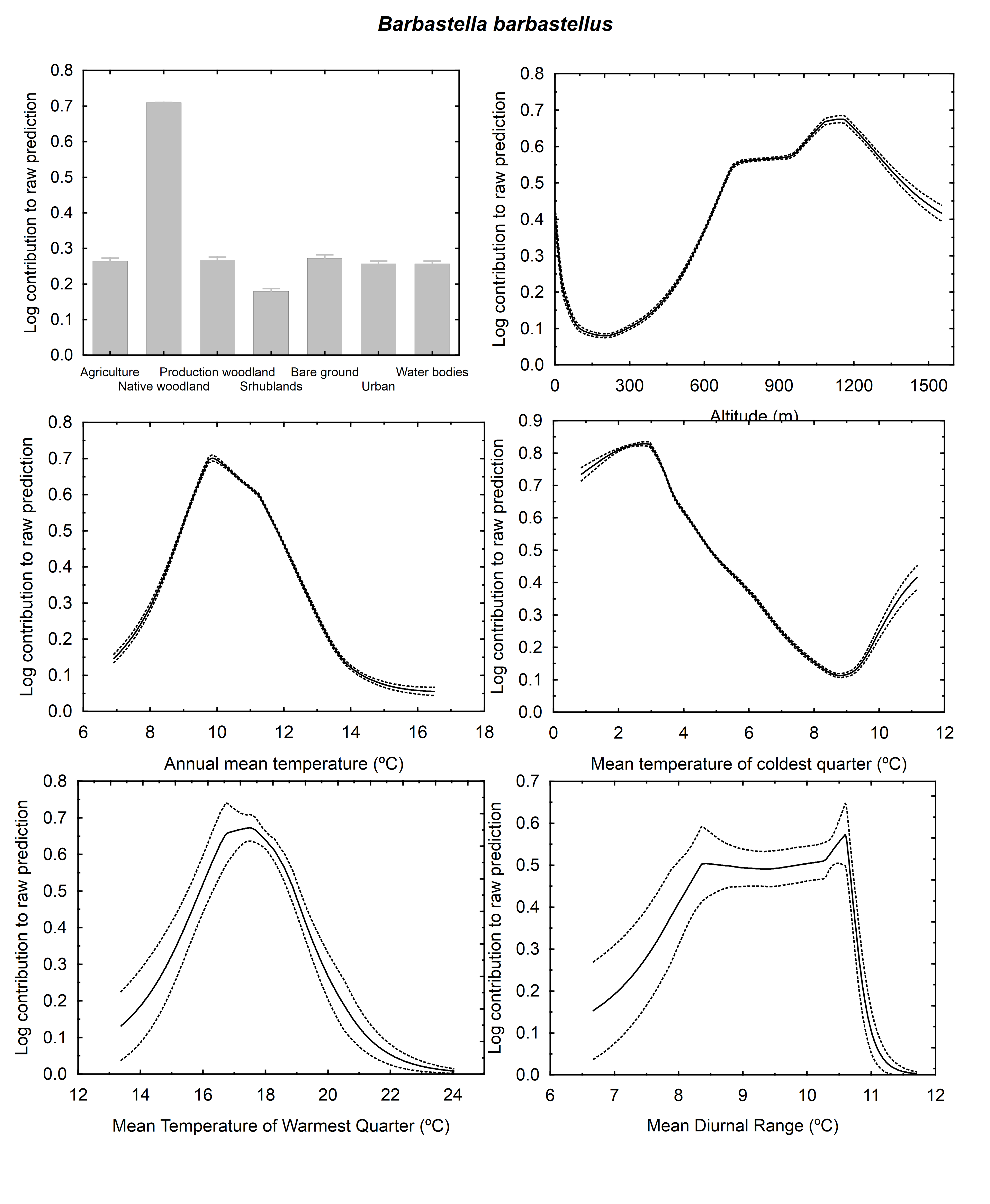

Supplement: Figure S9 — Response curves for the EGVs most related to the predicted distribution of Barbastella barbastellus . (TIF) [file pone.0087291.s009.tif]

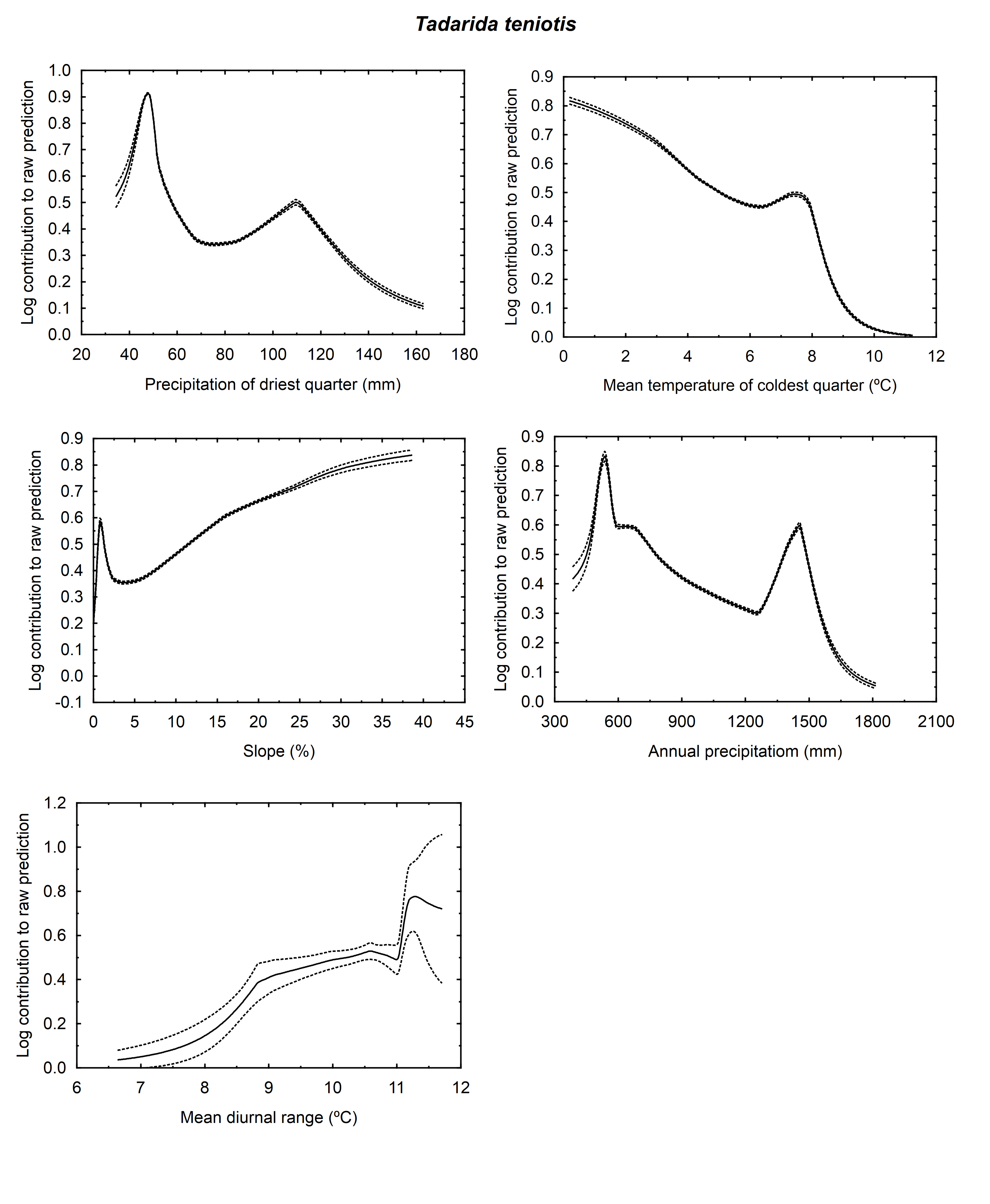

Supplement: Figure S10 — Response curves for the EGVs most related to the predicted distribution Tadarida teniotis . (TIF) [file pone.0087291.s010.tif]

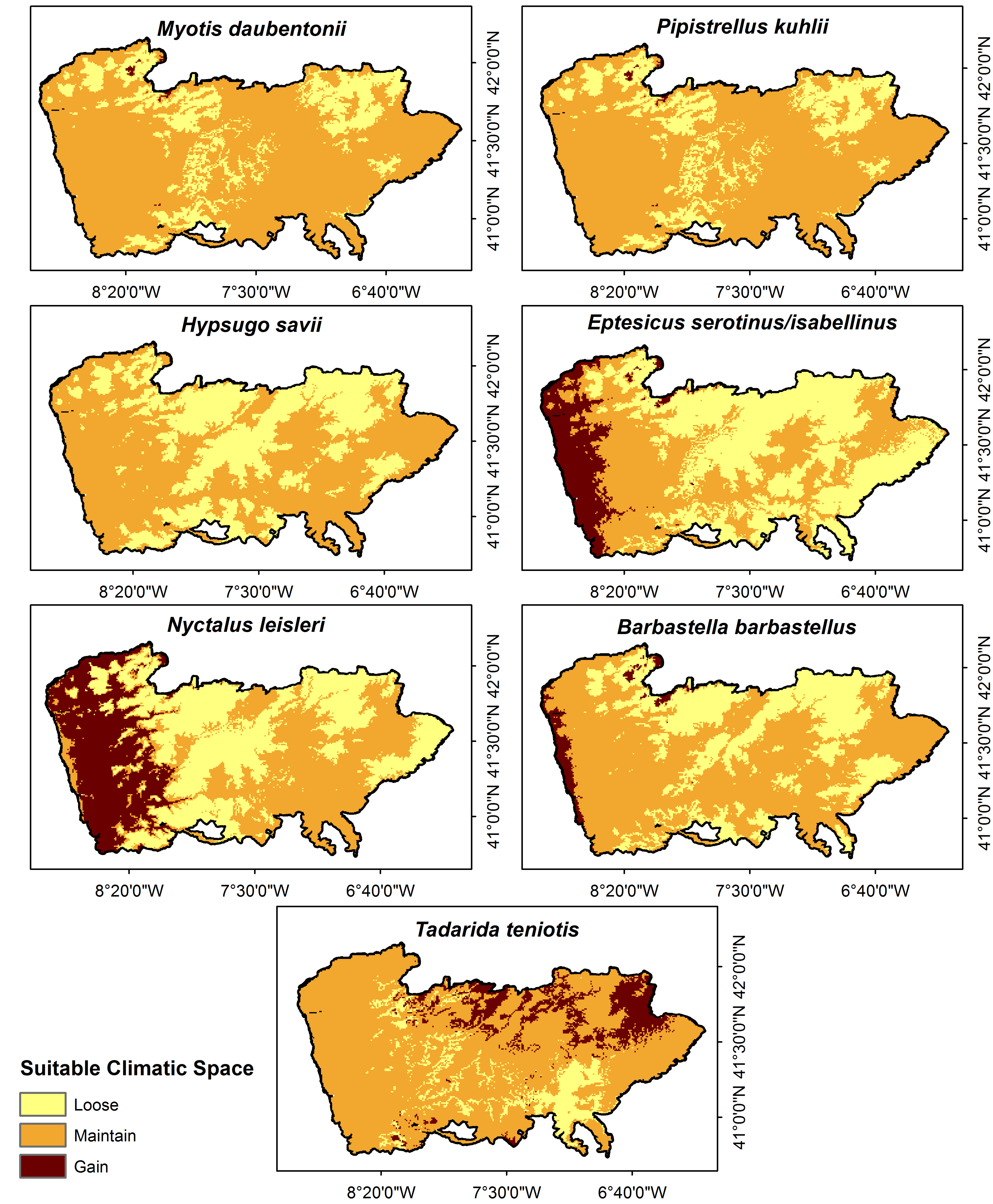

Supplement: Figure S11 — Areas where each target species is likely to gain, lose or maintain suitable climatic space under scenario A2a. (TIF) [file pone.0087291.s011.tif]

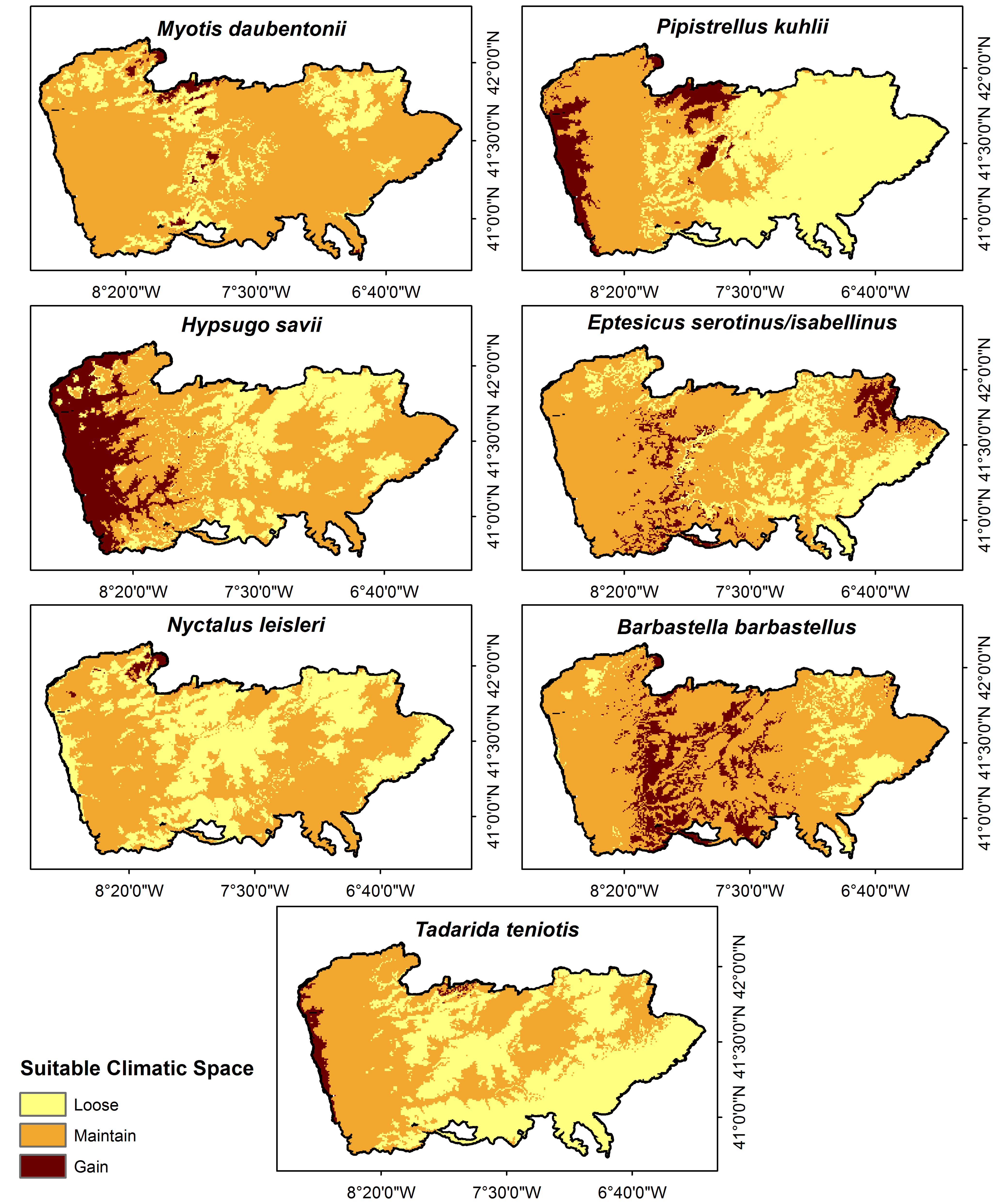

Supplement: Figure S12 — Areas where each target species is likely to gain, lose or maintain suitable climatic space under scenario B2a. (TIF) [file pone.0087291.s012.tif]

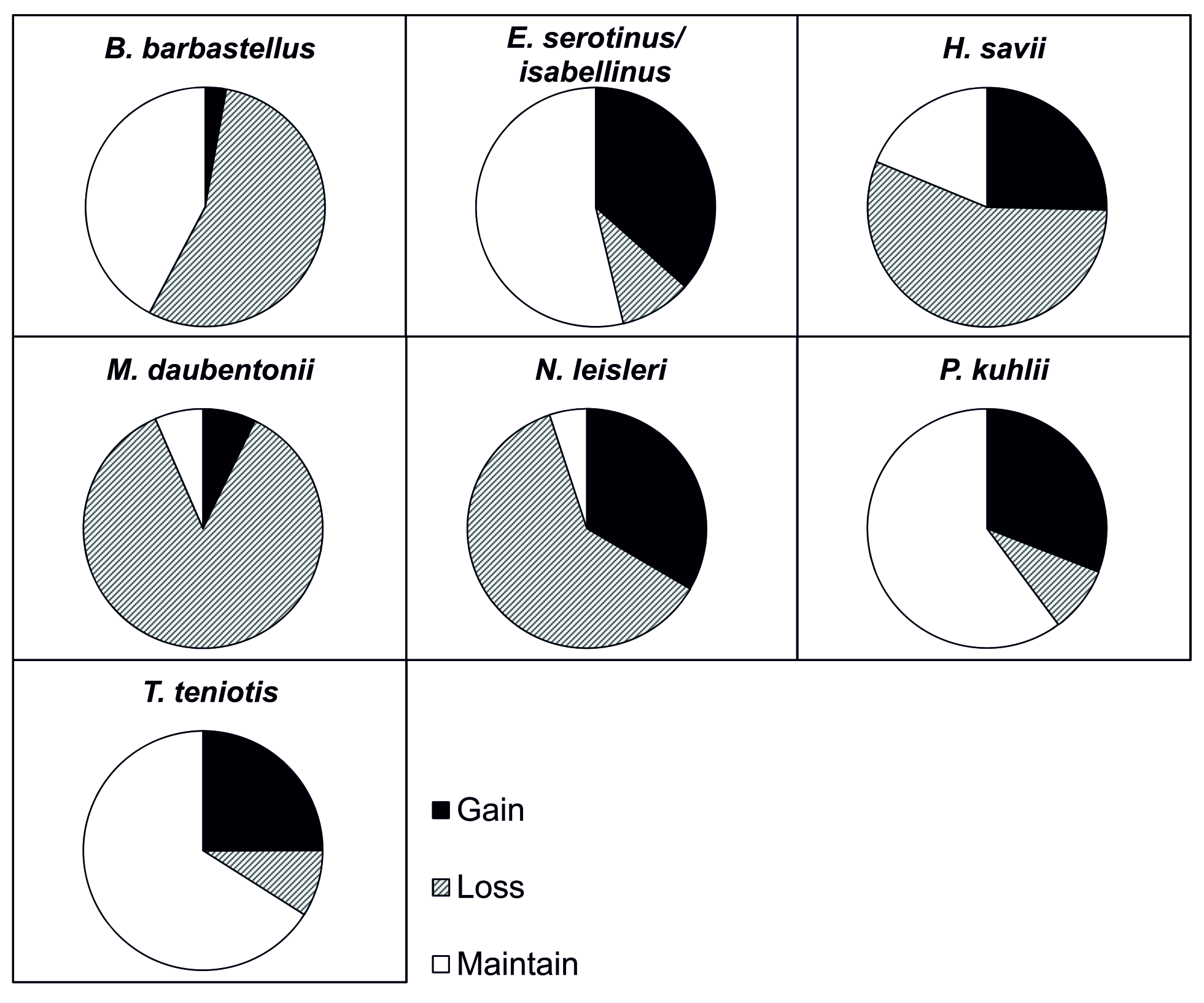

Supplement: Figure S13 — Proportion of area occupied by suitability class for each species. (TIF) [file pone.0087291.s013.tif]
