# Supplementary material for: Designing Optimized Multi-Species Monitoring Networks to Detect Range Shifts Driven by Climate Change: A Case Study with Bats in the North of Portugal
Source: PLoS One. 2014 Jan 27;9(1):e87291. doi: 10.1371/journal.pone.0087291 (PMC3903647; doi:10.1371/journal.pone.0087291)
Supplement: Table S1 — AUC values for Training and Test data for both Full and Climatic models. (DOCX) [file pone.0087291.s014.docx]

|  | Full | |  | Climatic | |
| --- | --- | --- | --- | --- | --- |
|  | Test Auc | Train Auc |  | Test Auc | Train Auc |
| Mdau | 0.8245 | 0.9251 |  | 0.8208 | 0.91 |
| Pkuh | 0.7835 | 0.8631 |  | 0.7588 | 0.8227 |
| Hsav | 0.7874 | 0.8923 |  | 0.7497 | 0.8576 |
| Eser/isa | 0.791 | 0.8809 |  | 0.7778 | 0.8435 |
| Nlei | 0.8094 | 0.8889 |  | 0.7733 | 0.8527 |
| Bbar | 0.8633 | 0.9253 |  | 0.8252 | 0.8773 |
| Tten | 0.8258 | 0.8927 |  | 0.8125 | 0.8628 |
