# Supplementary material for: Designing Optimized Multi-Species Monitoring Networks to Detect Range Shifts Driven by Climate Change: A Case Study with Bats in the North of Portugal
Source: PLoS One. 2014 Jan 27;9(1):e87291. doi: 10.1371/journal.pone.0087291 (PMC3903647; doi:10.1371/journal.pone.0087291)
Supplement: Table S2 — Predictor variables by order of relevance for each species for both Full and Climatic models. (DOCX) [file pone.0087291.s015.docx]

|  | Full |  | Climatic |
| --- | --- | --- | --- |
|  | Variables |  | Variables |
| Mdau | land cover, annual mean temperature, mean diurnal range, precipitation of driest quarter |  | annual mean temperature, mean diurnal range, precipitation of driest quarter, annual precipitation |
| Pkuh | annual precipitation, precipitation of driest quarter, slope, land cover |  | annual precipitation, precipitation of driest quarter, mean temperature of coldest quarter, mean diurnal range |
| Hsav | land cover, altitude, mean temperature of coldest quarter, annual mean temperature |  | annual mean temperature, mean temperature of coldest quarter, annual precipitation, mean temperature of warmest quarter |
| Eser/isa | land cover, altitude, slope, mean temperature of coldest quarter |  | mean temperature of coldest quarter, mean diurnal range, annual mean temperature, annual precipitation |
| Nlei | land cover, annual mean temperature, altitude, mean temperature of coldest quarter |  | annual mean temperature, mean temperature of coldest quarter, mean diurnal range, annual precipitation |
| Bbar | land cover, altitude, annual mean temperature, mean temperature of coldest quarter |  | annual mean temperature, mean temperature of coldest quarter, mean temperature of warmest quarter, mean diurnal range |
| Tten | precipitation of driest quarter, mean temperature of coldest quarter, slope, annual precipitation |  | precipitation of direst quarter, mean temperature of coldest quarter, annual precipitation, mean diurnal range |
