# Supplementary material for: Designing Optimized Multi-Species Monitoring Networks to Detect Range Shifts Driven by Climate Change: A Case Study with Bats in the North of Portugal
Source: PLoS One. 2014 Jan 27;9(1):e87291. doi: 10.1371/journal.pone.0087291 (PMC3903647; doi:10.1371/journal.pone.0087291)
Supplement: Table S3 — Percentage of the total number of cells that overlap is observed in both Full and Climatic and ENMtools results. (DOCX) [file pone.0087291.s016.docx]

|  | % | | | |  | Niche overlap | |  | Niche breadth | |
| --- | --- | --- | --- | --- | --- | --- | --- | --- | --- | --- |
|  | Uns | Sui | Clim | Full |  | I | D |  | Full | Clim |
| Mdau | 72 | 10 | 8 | 9 |  | 0.95 | 0.75 |  | 0.52 | 0.72 |
| Pkuh | 40 | 36 | 17 | 8 |  | 0.98 | 0.84 |  | 0.68 | 0.8 |
| Hsav | 55 | 25 | 13 | 6 |  | 0.98 | 0.84 |  | 0.65 | 0.75 |
| Eser/isa | 44 | 30 | 20 | 7 |  | 0.97 | 0.82 |  | 0.66 | 0.76 |
| Nlei | 53 | 25 | 17 | 5 |  | 0.98 | 0.83 |  | 0.66 | 0.78 |
| Bbar | 65 | 15 | 18 | 2 |  | 0.96 | 0.78 |  | 0.44 | 0.59 |
| Tten | 50 | 28 | 19 | 3 |  | 0.99 | 0.88 |  | 0.66 | 0.71 |
